# An Acrolein-Based Drug Delivery System Enables Tumor-Specific Sphingosine-1-Phosphate Targeting in Breast Cancer without Lymphocytopenia

**DOI:** 10.1158/2767-9764.CRC-25-0023

**Published:** 2025-06-18

**Authors:** Masayuki Nagahashi, Miki Komatsu, Sayaka Urano, Mamiko Kuroiwa, Yuria Takahashi, Koji Morimoto, Ambara R. Pradipta, Katsunori Tanaka, Yasuo Miyoshi

**Affiliations:** 1 Division of Breast and Endocrine Surgery, Department of Surgery, Hyogo Medical University, Nishinomiya, Japan.; 2 Department of Chemical Science and Engineering, School of Materials and Chemical Technology, Institute of Science Tokyo, Ookayama, Japan.; 3 Biofunctional Synthetic Chemistry Laboratory, RIKEN Pioneering Research Institute, Wako, Japan.; 4 Department of Human Health Science, Faculty of Human Sciences, Osaka International University, Moriguchi, Japan.

## Abstract

We developed a novel FTY720 prodrug (pro-FTY) that specifically inhibits sphingosine-1-phosphate signaling in cancer cells using a novel drug delivery system that reacts with acrolein. Our objective was to evaluate the efficacy and safety of pro-FTY in preclinical experiments. Ten breast cancer cell lines, two multidrug-resistant cell lines, and one normal mammary cell line were used to compare the IC_50_ values of pro-FTY with those of other drugs. Patient-derived organoids (PDO) were established and utilized for IC_50_ value comparisons. Drug efficacy was tested in mice bearing either syngeneic 4T1 cell tumors or patient-derived xenograft tumors, and blood analysis (including mass spectrometry) was performed. FTY720 and pro-FTY inhibited the survival of all breast cancer cell lines, including multidrug-resistant cells resistant to paclitaxel or doxorubicin. Unlike pro-FTY, FTY720 inhibited the survival of normal breast cell lines, suggesting that pro-FTY does not affect normal breast cells. Pro-FTY showed reproducible activity against multidrug-resistant PDOs, whereas paclitaxel and doxorubicin did not. Mass spectrometric analysis of pro-FTY–treated mice showed that FTY720 accumulated in tumors but was barely detectable in blood. Importantly, lymphocytopenia occurred in FTY720-treated mice but not in pro-FTY–treated mice. Furthermore, intravenous pro-FTY treatment significantly suppressed tumor growth in mice bearing patient-derived xenograft tumors generated from multidrug-resistant PDOs. In conclusion, pro-FTY inhibited breast cancer, including multidrug-resistant breast cancer, while avoiding lymphocytopenia, highlighting its clinical potential.

**Significance:**

Pro-FTY selectively inhibits sphingosine-1-phosphate signaling in cancer cells using a novel acrolein-responsive drug delivery system that reacts with acrolein. Pro-FTY does not inhibit normal cell growth, thus avoiding lymphocytopenia. Pro-FTY is effective against multidrug-resistant breast cancer with a unique mechanism of action, highlighting its translational and therapeutic potential.

## Introduction

Breast cancer is the most common cancer in women worldwide, and several patients succumb to death due to recurrent or metastatic disease (1, 2). According to a report based on the Surveillance, Epidemiology, and End Results database, in the United States, the overall mortality rate of breast cancer is 19.3 per 100,000 women (3). The incidence of breast cancer is 131.1 per 100,000 women; thus, approximately 15% of those who develop breast cancer die from it (3). Metastatic disease at initial diagnosis accounts for 6% of all cases, and the 5-year survival rate is ∼30% in those cases (3). Furthermore, nearly 10% of patients with nonmetastatic breast cancer at diagnosis die within 5 years due to subsequent metastasis or recurrence. Metastatic recurrent breast cancer is generally treated with systemic administration of anticancer drugs, but with repeated treatment, the cancer cells eventually develop drug resistance, resulting in mortality (4–6). Although new drugs [including cyclin-dependent kinase 4 and 6 inhibitors, PI3K and AKT inhibitors, PARP inhibitors, antibody–drug conjugates (ADC), and immune checkpoint inhibitors] are available for patients with metastatic breast cancer, and patient outcomes have improved, the progression-free survival of patients with metastatic breast cancer after repeated treatment remains dismal at only a few months. Each new treatment for metastatic breast cancer approved to date has revealed a unique resistance mechanism. For example, previous reports suggest that increased signaling in the PI3K pathway or mutations in the *RB1* or *TP53* genes may cause resistance to cyclin-dependent kinase 4 and 6 inhibitors (7). Resistance to PI3K and AKT inhibitors might occur due to specific gene alterations that activate the signaling pathways associated with the drug targets (8), whereas resistance to PARP inhibitors is believed to reflect reversion mutations or activation of other DNA-repair mechanisms (9). Decreased expression of proteins, such as the HER2 protein (the target of certain ADCs), in cancer cells results in drug resistance to these therapies (10). In addition, inadequate antigen recognition by T cells, impaired T-cell migration and/or infiltration, and reduced T-cell cytotoxicity may drive resistance to immune checkpoint inhibitors (11). Thus, developing new drugs with distinct mechanisms from those of conventional drugs is essential for avoiding drug resistance.

Sphingosine-1-phosphate (S1P) is a pleiotropic lipid mediator that participates in multiple cellular functions, including cell proliferation, migration, and survival (12, 13). S1P is produced intracellularly from sphingosine by two different sphingosine kinases (SphK1 and SphK2) and released from cells through S1P-specific transporters, in which it can act on five different S1P-specific cell-surface receptors (S1PR1–5) in autocrine and paracrine manners (14). Cancer cells often highly express SphK1 and produce high levels of S1P, which together promote cancer progression and metastases (15). S1P also drives cancer cell survival and drug resistance (16) and has attracted attention as a potential therapeutic target. S1P signaling inhibitors are expected to be effective against multidrug-resistant cancers because they inhibit S1P signaling, which distinguishes them mechanistically from existing drugs. Indeed, FTY720 (fingolimod), which is an S1P receptor modulator, has demonstrated a wide range of anticancer activity (17–20). Importantly, S1P potently guides lymphocytes from secondary lymphoid organs into the blood, and suppressing S1P signaling can result in lymphocytopenia (21, 22). Consequently, developing FTY720 and other S1P-signaling inhibitors as anticancer agents has been hampered by their immunosuppressive side effects (23). Targeted drug delivery systems (DDS) have transformed anticancer therapy, with applications in breast cancer (24). Examples of targeted DDSs include the ADCs, trastuzumab emtansine, trastuzumab deruxtecan, and sacituzumab govitecan, which have substantially improved treatment outcomes for patients with breast cancer and are currently used as standards of care (25–27). Thus, expanding the frontiers of DDSs may open up new therapeutic opportunities.

In this study, we developed a novel FTY720 prodrug (pro-FTY) using a DDS that targets acrolein. Acrolein is highly and specifically expressed in cancer cells, and it has recently attracted attention as a marker for intraoperative cancer and as a DDS target (28). Using this DDS, the prodrug pro-FTY was activated and only acted effectively against cancer cells while avoiding the adverse side effect of lymphocytopenia. We evaluated the efficacy of pro-FTY against breast cancer, including multidrug-resistant breast cancer, and its effect on lymphocytopenia *in vivo*.

## Materials and Methods

### Cell lines and standard culture conditions

The Breast Cancer p53 Hotspot Mutation Cell Panel (TCP-2010), consisting of the HCC38 (RRID: CVCL_1267), BT-549 (RRID: CVCL_1092), MDA-MB-468 (RRID: CVCL_0419), MDA-MB-175-VII (RRID: CVCL_1400), MDA-MB-361 (RRID: CVCL_0620), HCC70 (RRID: CVCL_1270), AU-565 (RRID: CVCL_1074), and SK-BR-3 (RRID: CVCL_0033) cell lines, was purchased from the ATCC, and each cell line was cultured according to the respective product sheet.

The human MCF-7 breast cancer cell line (RRID: CVCL_0031) was purchased from EMD Millipore. The cells were cultured in DMEM/F12 without phenol red (Sigma, catalog number D6434) containing 10% FBS (EMD Millipore, catalog number ES- 009-B), 2.5 mmol/L l-glutamine (EMD Millipore, catalog number TMS-002-C), 6 ng/mL insulin (Sigma, catalog number I-9278), and 1× antibiotic–antimycotic solution (Gibco, catalog number 15240096; ref. 29). MDA-MB-231 cells were cultured with atmospheric air (without CO_2_) in Leibovitz’s L-15 medium (Gibco, catalog number 11415-064) containing 10% FBS (Biowest, catalog number S1810-500) and 1× antibiotic–antimycotic solution (Gibco).

The MCF-10A cell line (a nontumorigenic epithelial cell line, RRID: CVCL_0598) was purchased from the ATCC. The cells were cultured in Mammary Epithelial Cell Growth Basal Medium, Phenol Red Free (Lonza, catalog number CC-3153) supplemented with components of the MEGM BulletKit (Lonza, catalog number CC-4136) and 100 ng/mL cholera toxin (FUJIFILM Wako Pure Chemical Corporation, catalog number 030-20621).

All cell lines obtained from the ATCC were authenticated via short tandem repeat analysis and passaged in our laboratory for fewer than 6 months after resuscitation. The MDA-MB-231R and MCF-7R cell lines used in multidrug-resistance experiments were not authenticated. All cell lines were routinely tested for *Mycoplasma* contamination using Mycoplasma Detection Kit (MycoStrip; InvivoGen, catalog number rep-mys-20). Except for the multidrug-resistance experiment, cells were cultured for up to 20 passages after thawing and used in all experiments. For multidrug-resistance experiments, 20 to 30 passages of the resistant cell lines were used.

### Establishment of multidrug-resistant cell lines

We established two multidrug-resistant cell lines (MDA-MB-231R and MCF-7R) from MDA-MB-231 and MCF-7 cells, respectively, by exposing them continuously to increasing concentrations of eribulin (Eisai Co., Ltd.; ref. 30). Initially, the MDA-MB-231 cells were exposed to 0.3 nmol/L eribulin, and this concentration was sequentially increased by 1.5-fold upon resistance acquisition until resistance at a final concentration of 656 nmol/L was reached.

The MCF-7 cells were exposed to an initial eribulin concentration of 1 nmol/L, and this concentration was sequentially increased by 1.5-fold upon resistance acquisition until resistance at a final concentration of 675 nmol/L was reached.

### Cell viability assay

The sensitivity of the cell lines to various drugs was evaluated using Cell Count Reagent SF (WST-8; Nacalai Tesque, catalog number 07553-44). Briefly, cells (2,000 cells/well in a 96-well plate) were cultured (24 hours) in 90 μL of culture medium (29, 30). Next, 10 μL of medium containing increasing concentrations of eribulin, doxorubicin (Tokyo Chemical Industry Co. Ltd., catalog number D4193), paclitaxel (Tokyo Chemical Industry, catalog number P1632), FTY720 (Cayman Chemical, catalog number 10006292), or pro-FTY was added to each well. After incubation for 96 hours, culture supernatants were collected for cryopreservation, and a mixture of 90 μL Dulbecco’s PBS (Nacalai Tesque, catalog number 14249-24) and 10 μL of Cell Count Reagent SF was added to each well. After additional 2 hours of incubation, the absorbance of each well was measured at 450 nm using a SpectraMax Plus 384 microplate reader (Molecular Devices), and the cell viability was determined. The absorbance of each sample was measured at least 3 times.

### Breast cancer tissue processing

Breast cancer tissues were obtained from surgical or biopsy specimens of patients at Hyogo Medical University Hospital. The collection and use of all specimens in this study were approved by the Institutional Review Board of Hyogo Medical University (approval number 3940). Written informed consent was obtained from all participants, and the study was conducted in accordance with the Declaration of Helsinki.

Upon acquisition, breast cancer tissues were cut into 1 to 3 mm^3^ pieces and washed thoroughly with DMEM [GlutaMAX Supplement (Gibco, catalog number 10569010) with 0.1% BSA (Sigma, catalog number A6003) and 1% penicillin–streptomycin (Nacalai Tesque, catalog number 26253-84); referred to as D-BSA]. Subsequently, the tissues were digested with 1 mg/mL collagenase (Gibco, catalog number 17101015) containing 10 μmol/L Y-27632 (AbMole BioScience, catalog number M1817) on a horizontal shaker at 37°C for 0.5 to 2 hours (31, 32). After additional washing and filtration, the cell suspension was centrifuged at 450 × *g* for 5 minutes at 8°C. In cases in which a visible red pellet was observed, the erythrocytes were lysed in 1 mL red blood cell lysis buffer (Roche, catalog number 11814389001) for 2 minutes at room temperature before adding 10 mL D-BSA and centrifuging at 450 × *g*. Each pellet was resuspended in 10 mg/mL ice-cold Cultrex Reduced Growth Factor Basement Membrane Extract (BME), Type 2 (Bio-Techne, catalog number 3533-005-02).

### Patient-derived organoid culture

Approximately 100 μL of BME–cell suspension was plated as multiple small drops per well in prewarmed 12-well suspension culture plates (Greiner, catalog number 665102) and solidified at 37°C for 30 minutes (31). Upon complete gelation, 750 μL of breast cancer organoid medium was added to each well, and the plates were transferred to a humidified incubator (37°C/5% CO_2_). The medium was changed every 4 days, and the organoids were passaged every 1 to 4 weeks.

### Drug sensitivity testing

Organoids were dissociated into single cells using TrypLE Express (Gibco, catalog number 12605-010), strained through a 70-μm cell strainer, and seeded in 12-well culture plates (32). The organoids were harvested 5 to 7 days after seeding, diluted to a concentration of 30 to 200 organoids/μL in BME, replated in 96-well plates (10 μL of BME/well), and cultured for 2 to 4 days. The culture medium was then replaced with fresh medium, and 10 μL of medium containing increasing concentrations of doxorubicin, paclitaxel, FTY720, or pro-FTY was added to each well. Subsequently, the organoids were incubated for 5 days. To measure adenosine triphosphate as a proxy for viable cells, 50 μL of culture medium was removed, and BME drops were suspended in 50 μL CellTiter-Glo 3D Reagent (Promega, catalog number G9681). Each resulting suspension was transferred to a separate well in a 96-well, white round-bottom plate (Corning, catalog number 3789A), shaken for 5 minutes, and incubated for 15 minutes at room temperature, after which luminescence was measured using a GloMax 96 microplate reader (Promega). Each condition was tested with triplicate samples.

### Acrolein staining

To detect acrolein in breast cancer and normal breast tissues, they were stained as previously reported (33). Briefly, fresh tissue samples were cut to an appropriate size with a razor blade, immersed in 20 μmol/L Click-to-Sense (CTS) probe solution in 15-mL conical tubes for 5 minutes at room temperature, and then rinsed 3 times with PBS. The nuclei of the resulting tissues were stained with Hoechst 33342 and Hoechst 33258 (Dojindo). The tissue samples were transferred to separate glass-bottomed dishes (Thermo Scientific Nunc) and placed under a fluorescence microscope (Keyence, BZ-X710) equipped with an optical-sectioning algorithm system (to obtain clear images without fluorescence blurring). Fluorescence images of the whole tissues were taken under low power.

To detect acrolein in patient-derived organoids (PDO), they were cultured on eight-well glass Millicell EZ slides (Merck Millipore, catalog number PEZGS0816), incubated in a 2 μmol/L solution of CTS probe for 15 minutes at room temperature, and rinsed thrice with PBS. After incubation with 0.5% Triton X-100 (Merck Millipore, catalog number SLCK9397) and subsequent fixation with 4% paraformaldehyde (Bioenno Tech, 006775-1L) at room temperature (10 minutes per step), the organoids were mounted with VECTASHIELD HardSet with 4′,6-diamidino-2-phenylindole (Vector Laboratories, Inc., catalog number H-1500) and placed under a BZ-X710 fluorescence microscope (Keyence).

### Animal models

All animal studies were conducted in the Animal Research Facility of Hyogo Medical University in accordance with our institutional guidelines. The animals were bred and maintained in a pathogen-free environment, and all procedures were approved by the Hyogo Medical University Institutional Animal Care and Use Committee, which is accredited by the Association for Assessment and Accreditation of Laboratory Animal Care.

Female BALB/c mice (RRID: IMSR_JCL:JCL:MIN-0005) were obtained from CLEA Japan, Inc. and used to establish the syngeneic 4T1 breast cancer cell implantation model. 4T1 breast cancer cells (RRID: CVCL_0125; 5 × 10^5^ cells in 50 μL of a 1:1 mixture of medium and BME) were surgically implanted in the upper mammary fat pad under direct visualization, as described previously (34). Tumor dimensions were measured with calipers every 3 days, and total tumor volumes were estimated using the cylindrical volume formula. Tumor-bearing mice were randomized 6 days after implantation before treatment with saline, FTY720, or pro-FTY. Based on the solubility of pro-FTY720, FTY720 or pro-FTY was administered via tail vein injection at a dose of 240 nmol/day [equivalent (eq) to FTY720 4.1 mg·kg^–1^·day^–1^, pro-FTY 6.8 mg·kg^–1^·day^–1^], which is comparable with previous reports (35), in a solution consisting of Tween 80, DMSO, and saline (1:4:95, v/v/v) on days 6, l9, and 12 after implantation. The mice were euthanized via exsanguination on day 13 or 15 after implantation. Blood was collected, and tumors were excised, weighed, fixed in formalin, and embedded in paraffin or frozen in liquid nitrogen. IHC analysis of phospho-Stat3 (pStat3) was performed on paraffin-embedded tumor tissues following histologic assessments, as previously described (36). Briefly, antigen retrieval was performed in target-retrieval solution with a pH 9 Tris-based antigen unmasking solution (Vector Laboratories), and a primary antibody [pStat3 (Tyr705); Cell Signaling Technology, Inc., RRID: AB_2491009] was used at a 1:100 dilution. Plasma levels of FTY720, phosphorylated FTY720 (FTY720-P), and pro-FTY were quantified via mass spectrometry (Shimadzu Techno-Research, Inc.). Additionally, complete blood counts were performed with whole blood samples at Oriental Yeast Co., Ltd. Blood T-cell subsets were analyzed via flow cytometry using the following antibodies: FITC-conjugated anti-CD4 mAb (BioLegend, RRID: AB_312690), PE-conjugated anti-CD8α mAb (BioLegend, RRID: AB_312746), and allophycocyanin-conjugated anti-CD3 mAb (BioLegend, RRID: AB_2561455). Stained cells were detected using a BD LSR-Fortessa X-20 cell analyzer (BD Biosciences) and analyzed using BD FACSDiva software (RRID: SCR_001456), as described (37). CD4^+^ T lymphocytes were defined as CD3^+^CD4^+^ cells, whereas CD8^+^ T lymphocytes were defined as CD3^+^CD8^+^ cells, according to a previous report (37). Serum IL-6 levels were determined using LBIS TM Mouse IL-6 ELISA Kit (FUJIFILM Wako Pure Chemical Corporation) as per the manufacturer’s instructions.

Female NOD/Shi-scid, IL-2RγKO Jic mice were obtained from In-Vivo Science, Inc. to establish a mouse patient-derived xenograft (PDX) breast cancer implantation model. PDO breast cancer cells (8 × 10^5^ cells in 50 μL of a 1:1 mixture containing medium and BME) were surgically implanted in the lower mammary fat pad under direct visualization, as described (23). The resulting tumors were serially passaged using 1 to 2 mm^3^ tumor fragments. Tumor dimensions were measured with calipers twice/week, and total tumor volumes were estimated using the cylindrical volume formula. Tumor-bearing mice were randomized 28 days after implantation before treatment with saline or pro-FTY. Pro-FTY was administered via tail vein injection at a dose of 240 nmol/day in a solution of Tween 80, DMSO, and saline (1/4/95, v/v/v) 5 times/week for 3 weeks. The mice were euthanized by exsanguination on day 21 after the initial injection. Blood was collected; and tumors were excised, weighed, fixed in formalin, and embedded in paraffin or frozen in liquid nitrogen. Additionally, biochemical serum tests were performed by Oriental Yeast Co., Ltd.

### Statistical analysis

Statistical analysis was performed using an unpaired, two-tailed Student *t* test when comparing two groups, and ANOVA followed by *post hoc* testing was used for multiple comparisons (GraphPad Prism, RRID: SCR_002798). A *P* value of <0.05 was considered to indicate a significant difference. Experiments were repeated at least three times with triplicate samples, with consistent results. *In vivo* experiments were repeated three times, and each experimental group consisted of at least five mice.

### Pro-FTY synthesis

**Figure 1:**
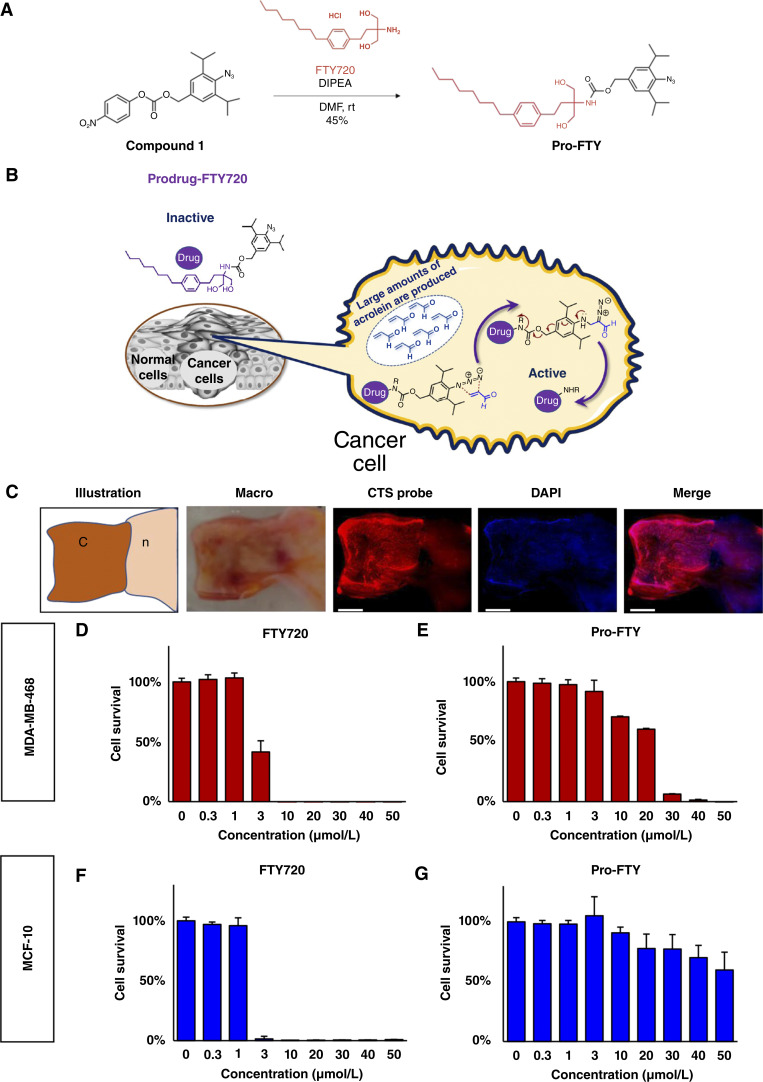
Pro-FTY, a novel anticancer prodrug that is converted to its active form (FTY720) after reacting with acrolein, which is overproduced in breast cancer cells. **A,** Synthesis of pro-FTY. FTY720 was reacted with compound **1** to synthesize pro-FTY. **B,** Schematic representation of the reaction of pro-FTY with acrolein in cancer cells to produce the active drug, FTY720, which can block S1P signaling. **C,** Acrolein expression in breast cancer tissue and normal breast tissue. Fluorescence microscopy images of acrolein expression in cancer (c) tissue and normal (n) breast tissue labeled with the CTS probe (red) and 4′,6-diamidino-2-phenylindole (DAPI; blue). Scale bars, 100 nm. **D–G,** Dose–response bar graphs for each drug in breast cancer and normal mammary cell lines. The IC_50_ values of FTY720 (**D**) and pro-FTY (**E**) were determined using the MDA-MB-468 breast cancer cell line by performing WST-8 assays, and the IC_50_ values of FTY720 (**F**) and pro-FTY (**G**) were determined in the noncancer, mammary epithelial cell line, MCF-10A.

Compound **1** (Fig. 1A) was synthesized as reported previously (38). FTY720 (95.0 mg, 0.28 mmol, 1.1 eq) was added to a mixture of compound **1** (100 mg, 0.25 mmol, 1.0 eq) and N,N-diisopropylethylamine (219 μL, 1.3 mmol, 5.5 eq) dissolved in dimethylformamide [2.5 mL, (**1**) = 0.1 mol/L] at ambient temperature. The reaction mixture was stirred at an ambient temperature for 20 hours. Dimethylformamide was concentrated to dryness under reduced pressure. The obtained residue was purified via column chromatography on silica gel [CHCl_3_/methanol (CHCl3 only to 10:1)] to yield the desired **pro-FTY** compound as a white solid (63.7 mg, 45%). ^1^H nuclear magnetic resonance (NMR) spectra were recorded at 400 MHz using CDCl_3_ as the solvent. The ^1^H NMR spectra showed the following resonances: δ 7.12 (s, 2H), 7.07 (d, *J* = 1.6 Hz, 4H), 5.30 (s, 1H), 5.04 (s, 2H), 3.92 (dd, *J* = 11.4, 6.1 Hz, 2H), 3.69 (dd, *J* = 11.6, 6.6 Hz, 2H), 3.35 (hept, *J* = 6.8 Hz, 2H), 3.12 (s, 2H), 2.62–2.49 (m, 4H), 1.94–1.86 (m, 2H), 1.36–1.18 (m, 22H), and 0.91–0.83 (m, 3H). ^13^C NMR spectra were recorded at 101 MHz using CDCl_3_ as the solvent. The ^13^C NMR spectra showed the following resonances: δ 143.51, 140.77, 138.48, 135.34, 134.43, 128.55, 128.08, 124.00, 66.96, 66.63, 59.58, 35.53, 31.89, 31.57, 29.48, 29.34, 29.27, 29.04, 28.84, 23.46, 22.67, and 14.11. Electrospray ionization–high-resolution mass spectrometry mass:charge ratios were calculated for C_33_H_50_N_4_NaO: [(M+Na)^+^], 589.3724; found, 589.3732.

### Data availability

The datasets used during the current study are available from the corresponding author on reasonable request.

## Results

### Released FTY720 reacted with acrolein generated in breast cancer cells

We developed pro-FTY, a novel cancer-specific S1P signaling inhibitor, utilizing a novel DDS that reacts with acrolein in cancer cells to generate the active drug, FTY720 (Fig. 1A and B). Using surgical specimens, we confirmed that acrolein was abundant in breast cancer tissues and was barely expressed in normal breast tissues (Fig. 1C). Both FTY720 and pro-FTY inhibited the survival of MDA-MB-468 breast cancer cells (Fig. 1D and E). Whereas FTY720 reduced cell survival more potently in normal MCF-10A breast epithelial cells than in MDA-MB-468 cells (Fig. 1F), pro-FTY did not inhibit MCF-10A cell survival, even at concentrations exceeding the IC_50_ value for MDA-MB-468 cells (Fig. 1G). Lower acrolein production in normal cells led to insufficient pro-FTY activation and low toxicity in MCF-10A cells.

### Pro-FTY inhibited the survival of all breast cancer cell lines examined at certain concentrations

**Figure 2:**
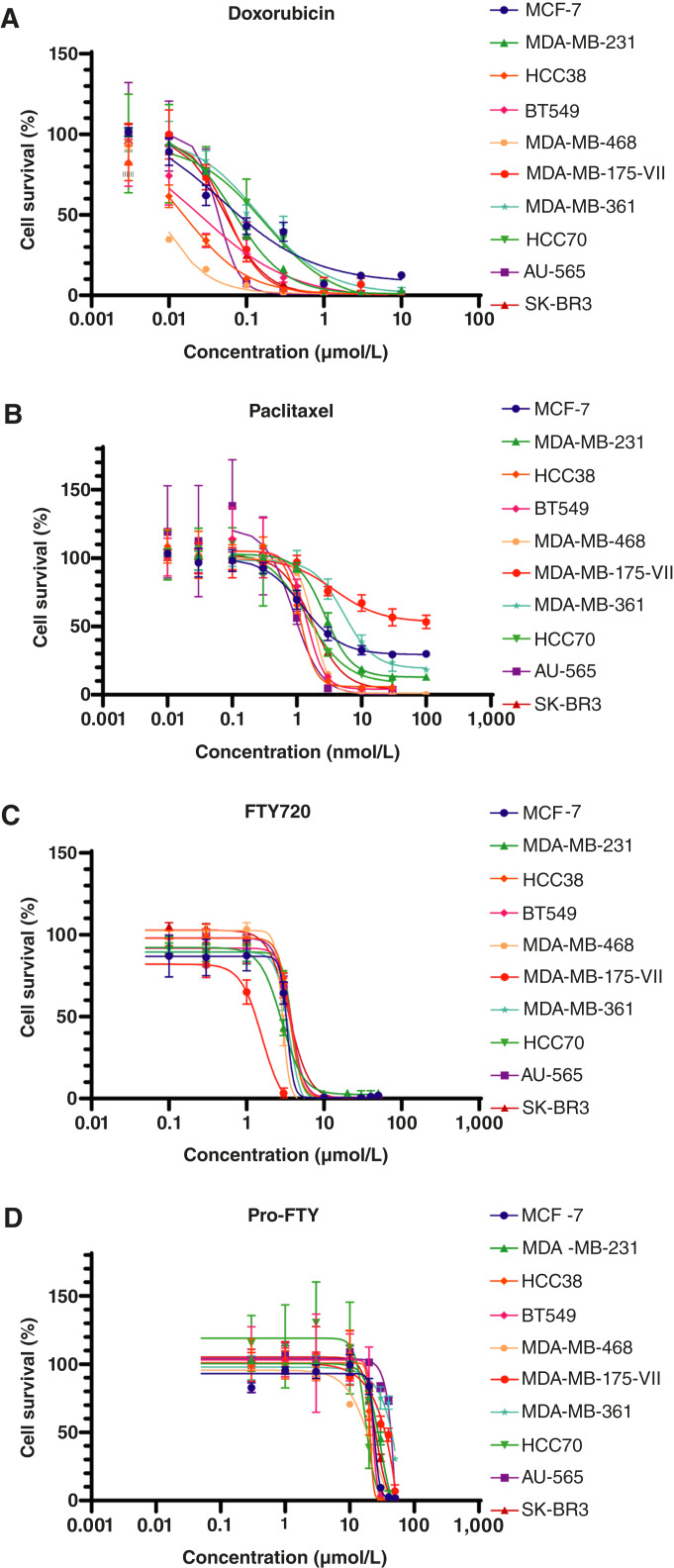
Dose–response curves for each drug in 10 breast cancer cell lines. **A–D,** Dose–response curves and IC_50_ values for doxorubicin (**A**), paclitaxel (**B**), FTY720 (**C**), and pro-FTY (**D**) were determined in each cell line by performing WST-8 assays.

We next tested the effects of FTY720 and pro-FTY on 10 different breast cancer cell lines (Fig. 2). The sensitivities of these 10 cell lines to doxorubicin and paclitaxel were measured, and their IC_50_ values were compared. The IC_50_ values for doxorubicin ranged from 0.001 to 1 μmol/L (Fig. 2A). Furthermore, the IC_50_ values for paclitaxel mostly ranged from 0.1 to 10 nmol/L. However, more than 50% of MDA-MB-175-VII cells survived paclitaxel exposure, even at 100 nmol/L (Fig. 2B).

In contrast, FTY720 consistently inhibited the survival of all cell lines tested, with IC_50_ values ranging from 1 to 10 μmol/L (Fig. 2C). Although pro-FTY required higher concentrations than FTY720 (because of a modification of the active amine moiety), its IC_50_ value fell mainly within a narrow range of 10 to 30 μmol/L. Moreover, like FTY720, pro-FTY showed consistent inhibition of cell survival (Fig. 2D).

### Pro-FTY consistently inhibited the growth of multidrug-resistant breast cancer cell lines

**Figure 3:**
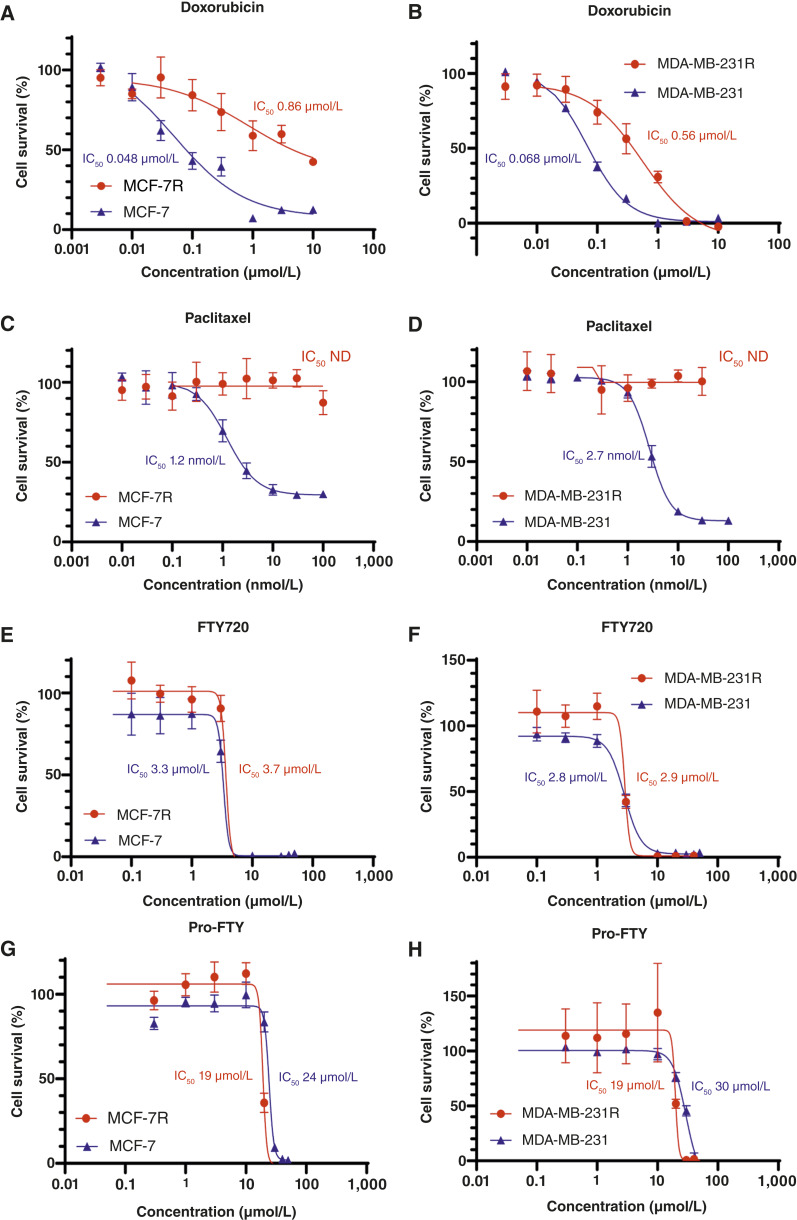
Dose–response curves for the indicated drugs in multidrug-resistant breast cancer cell lines. **A–H,** Dose–response curves and IC_50_ values of doxorubicin (**A** and **B**), paclitaxel (**C** and **D**), FTY720 (**E** and **F**), and pro-FTY (**G** and **H**) were determined in MCF-7 and MCF-7R cells (**A** and **C–E**) and MDA-MB-231 and MDA-MB-231R cells (**B**, **D**, **F**, and **H**) by performing WST-8 assays. ND, not determined.

Given the consistent efficacy of pro-FTY against all breast cancer cell lines tested, we evaluated its efficacy against multidrug-resistant breast cancer cell lines generated by long-term culture with anticancer drugs (Fig. 3). The IC_50_ value of doxorubicin for the multidrug-resistant breast cancer cell lines MCF-7R and MDA-MB-231R were >100-fold or >10-fold higher than that for the parental MCF-7 cell line, respectively (Fig. 3A and B). Furthermore, paclitaxel did not inhibit the survival of either cell line, even at high concentrations (Fig. 3C and D). In contrast, FTY720 consistently inhibited MCF-7R and MDA-MB-231R cell survival, with IC_50_ values comparable with that observed with the parental MCF-7 and MDA-MB-231 cell lines (Fig. 3E and F). Similarly, pro-FTY consistently inhibited the survival of MCF-7R and MDA-MB-231R cells, with IC_50_ values comparable with those found with the parental cell lines MCF-7 and MDA-MB-231 (Fig. 3G and H), respectively.

### Pro-FTY inhibited the growth of multidrug-resistant breast cancer PDOs

**Figure 4:**
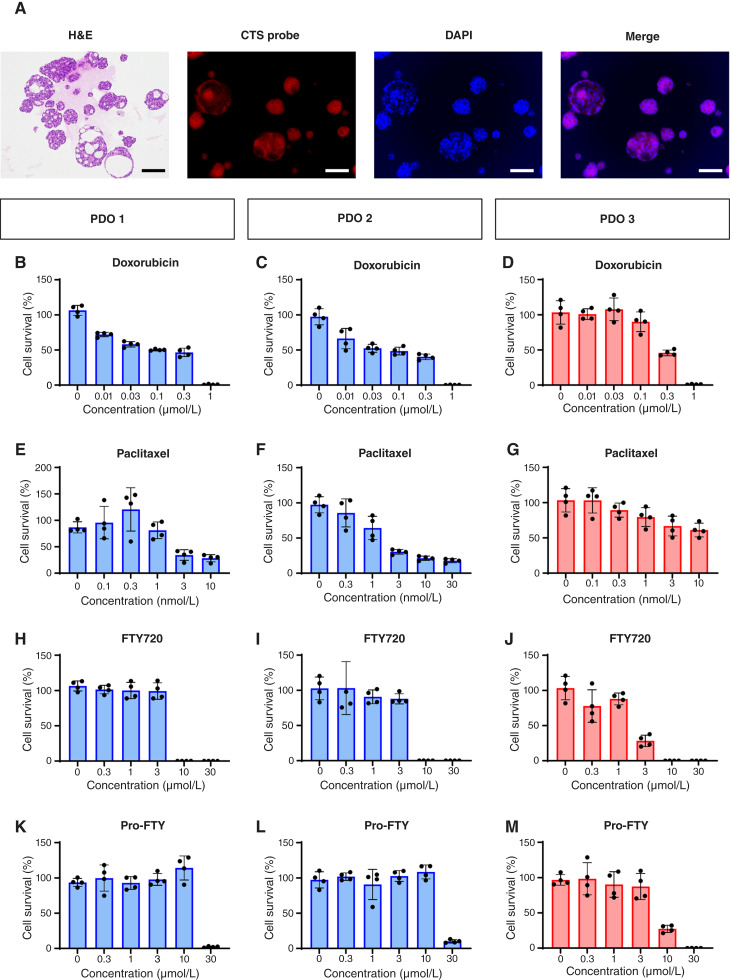
Effects of pro-FTY and other drugs on PDOs. **A,** PDOs stained with hematoxylin and eosin (H&E), the CTS probe (red), and 4′,6-diamidino-2-phenylindole (DAPI; blue), along with a merged image (merge). Scale bars, 100 µm. **B–M,** Effects of doxorubicin (**B–D**), paclitaxel (**E** and **F**), FTY720 (**H–J**), and pro-FTY (**K–M**) were determined with PDOs 1–3 by performing CellTiter-Glo 3D cell viability assays. PDOs 1 and 2 were generated from patients with treatment-naïve breast cancer, and PDO 3 was generated from a biopsy specimen from a patient acquired multidrug resistance by the end of treatment.

We then tested the efficacy of pro-FTY using organoids prepared from breast cancer tissues obtained from patients treated at our hospital (Fig. 4). Staining the breast cancer PDOs with a CTS probe confirmed that they expressed high levels of acrolein (Fig. 4A). PDOs 1 and 2 (used for drug sensitivity testing) were derived from treatment-naïve patients with breast cancer, and PDO 3 was generated using a biopsy specimen from a patient who developed multidrug resistance by the end of treatment (Fig. 4B–M). Doxorubicin had a lower effect on PDO 3 than on PDOs 1 and 2 (Fig. 4B–D).

Paclitaxel was less effective against PDO 3 than against PDOs 1 and 2 (Fig. 4E–G). In contrast, FTY720 inhibited the growth of the multidrug-resistant PDO 3 at lower concentrations than required for PDOs 1 and 2 (Fig. 4H–J). Interestingly, pro-FTY also inhibited PDO 3 growth at lower concentrations than required for PDOs 1 and 2 (Fig. 4K–M).

### Pro-FTY exerted antitumor effects *in vivo* while avoiding lymphocytopenia

**Figure 5:**
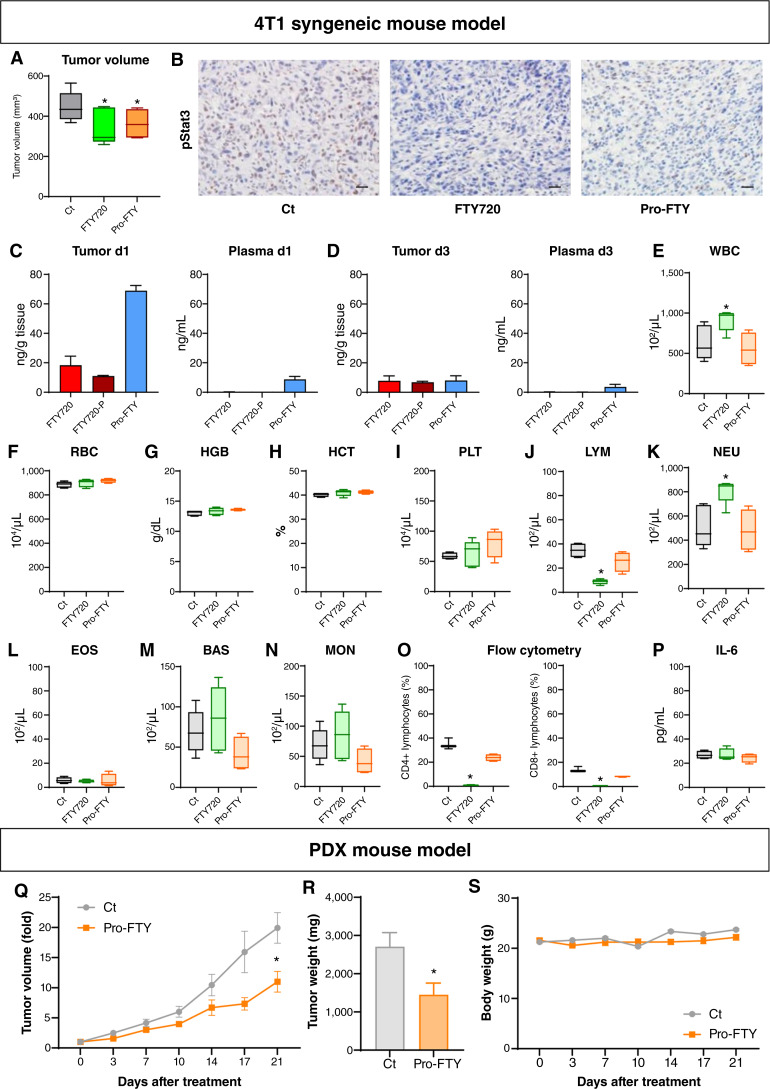
Pharmacokinetics of pro-FTY and its effect on lymphocytes and tumor suppression in mice. **A,** BALB/c and control (Ct) mice implanted with the 4T1 breast cancer cell line were treated three times with FTY720 and pro-FTY, and tumor volumes were measured. **B,** pStat3 expression in representative tumors from BALB/c mice administered the 4T1 breast cancer cell line after treatment with FTY720 or pro-FTY. Scale bars, 25 μm. **C** and **D,** Pro-FTY, FTY720, and FTY-P levels in tumor and plasma samples from BALB/c mice were quantified by mass spectrometry on d1 (**C**) and d3 (**D**) after the last pro-FTY drug administration. **E–N,** Blood counts were performed the day after the last dose of drug was administered: white blood cells (WBC; **E**), red blood cells (RBC; **F**), hemoglobin (HGB; **G**), hematocrit (HCT; **H**), platelets (PLT; **I**), lymphocytes (LYM; **J**), neutrophils (NEU; **K**), eosinophils (EOS; **L**), basophils (BAS; **M**), and monocytes (MON; **N**). **O,** CD4^+^ and CD8^+^ lymphocyte abundances were measured by flow cytometry. **P,** Serum IL-6 levels were determined by performing ELISAs. **Q** and **R,** With the PDX mouse model, the effect of pro-FTY was evaluated by measuring tumor volumes (**Q**) over time and tumor weights (**R**) when the mice were euthanized. *, *P* < 0.05 vs. control. **S,** Body weights of the mice in each group during the treatment period.

We developed a mouse model to evaluate the antitumor effects and pharmacokinetics of pro-FTY and determine whether administering it as a prodrug could avoid the side effect of lymphocytopenia (Fig. 5). In a syngeneic BALB/c mouse model implanted with 4T1 breast cancer cells, intravenous pro-FTY administration significant suppressed tumor growth (*P* < 0.05 vs. controls), comparable with FTY720 (Fig. 5A). IHC staining for pStat3 (part of the S1P signaling pathway) indicated that FTY720 or pro-FTY treatment lowered tumor-cell pStat3 expression more than control treatment (Fig. 5B). Intracellularly, pro-FTY can react with acrolein to cleave the protecting group and release the active form FTY720, which is further phosphorylated intracellularly to form FTY720-P. The concentrations of pro-FTY, FTY720, and FTY720-P in tumors and plasma at 1 and 3 days after pro-FTY administration (d1 and d3, respectively) were determined via mass spectrometry (Fig. 5C and D). Higher pro-FTY concentrations were detected in tumors than in plasma on d1, suggesting that the prodrug was converted to FTY720 and FTY720-P in the tumors (Fig. 5C). By d3, pro-FTY tumor levels had decreased significantly, whereas the decreases in FTY720 and FTY720-P levels were more gradual, indicating that pro-FTY was slowly converted to FTY720 and FTY720-P in tumors. Moreover, FTY720 and FTY720-P remained in the tumors for longer durations than pro-FTY (Fig. 5D). Pro-FTY was detectable in plasma on d1 and d3, whereas FTY720 and FTY720-P were not (Fig. 5C and D).

Using the same mouse model, complete blood counts were determined 1 day after FTY720 or pro-FTY administration. Complete blood counts were also determined for tumor-bearing control mice (Fig. 5E–N). Importantly, lymphocytopenia was not observed in pro-FTY–treated mice or control mice, whereas it was observed in FTY720-treated mice (Fig. 5J). Indeed, further flow cytometric analysis revealed that both CD4^+^ and CD8^+^ T lymphocytes were significantly less abundant in FTY720-treated mice but relatively preserved in pro-FTY–treated mice (Fig. 5O). Neutrophil and white blood cell levels increased after FTY720 treatment (Fig. 5E and J). Considering that serum abundance of IL-6 (an inflammatory cytokine) did not change in the FTY720-treated group (Fig. 5P), the increased abundance of neutrophils and white blood cells was not due to infection or inflammation but reflected a complementary biological response in which a marked decrease in lymphocytes was compensated for by neutrophils and other cells (Fig. 5E and J). No significant differences were observed in red blood cells, hematocrit percentages, or platelets among the three groups, except that hemoglobin was slightly elevated in the pro-FTY group (Fig. 5F–I). After treatment, hematoxylin and eosin–stained specimens of heart, kidney, liver, lung, and spleen were prepared for pathologic examination, and there was no evidence that adverse events occurred in the FTY720 and pro-FTY groups when compared with the control group (Supplementary Fig. S1A).

Finally, the efficacy of pro-FTY was tested using a PDX mouse model generated from multidrug-resistant PDOs (Fig. 5Q–S). Pro-FTY significantly suppressed the growth of the multidrug-resistant PDOs, based on lower tumor volumes (*P* < 0.05, Fig. 5Q) and tumor weights (*P* < 0.05, Fig. 5R) than found in untreated control mice. Throughout the course of treatment, body weights in the pro-FTY group were not significantly different than those in the control group, and no signs of morbidity or abnormal behavior (including activity levels, grooming, and distress signals) were observed (Fig. 5S).

## Discussion

Drug resistance is a major challenge in cancer treatment, and most patients with recurrence and/or metastasis eventually develop resistance to anticancer drugs during treatment, resulting in disease progression and death (4–6). As a sphingolipid mediator, S1P can promote cancer progression and mediate drug resistance (39). The S1P signaling pathway has long attracted attention as a therapeutic target for cancer (40). However, S1P is essential for lymphocyte egress from secondary lymphoid organs into the blood, and inhibiting S1P signaling significantly reduces the number of lymphocytes in the blood, resulting in immunosuppressive side effects (13, 14). Thus, systemic anti–S1P signaling therapy is currently unsuitable as an anticancer treatment. In this study, pro-FTY (the first S1P-targeting inhibitor that does not cause lymphocytopenia) was administered using a novel DDS in which the prodrug is converted to the active drug after interacting with acrolein, which is highly expressed in cancer cells. Treatment with pro-FTY showed consistent inhibitory effects on all types of breast cancer cells tested, including multidrug-resistant strains. Importantly, this effective treatment was achieved without evidence of lymphocytopenia.

Using an immunocompetent syngeneic implantation model involving BALB/c mice implanted with 4T1 mouse breast cancer cells, we confirmed that pro-FTY was selectively activated in cancer cells and that the released FTY720 exerted an antitumor effect without eliciting lymphocytopenia. Xenograft tumors derived from breast cancer cell lines and PDX mouse models are not suitable for assessing lymphocytopenia because they use immunocompromised mice lacking T cells, making subsequent verification with an immunocompetent syngeneic mouse model necessary (41). With the syngeneic mouse model developed in this study, no abnormalities were observed in terms of red blood cells or platelets other than white blood cells, and biochemical testing showed normal results. Following pro-FTY treatment, the mice remained healthy, and no weight loss or adverse effects were observed. Developing S1P-targeted therapy without immunosuppressive side effects using a DDS would represent a major step forward in overcoming one of the key barriers in S1P translational research (40).

FTY720 is already approved for treating multiple sclerosis (14). However, clinical results have raised concerns about its off-target effects (including bradycardia, macular edema, elevated liver enzymes, and an increased risk of infection due to lymphocyte depletion) when considering its use for cancer treatment. In contrast, treatment with pro-FTY might reduce or eliminate these side effects; FTY720 functions in cancer cells through a DDS that targets acrolein. As noted above, after pro-FTY treatment, the mice remained in good health and showed no significant organ damage, suggesting that the acrolein-targeted DDS may circumvent the adverse effects of FTY720.

In this study, we utilized a DDS that reacts with acrolein [which is highly and specifically expressed in breast cancer cells (33)] to activate FTY720 by releasing the attached protective group (33). As acrolein is highly expressed in breast cancer tissues, the CTS probe is currently being used diagnostically to detect cancer cells in surgical margins, and a clinical trial is underway to explore its intraoperative use (42). One feature of the DDS is that the reaction between the azide group of pro-FTY and acrolein can immolate the benzyl carbamate linker, with subsequent release of the active drug. This azide–acrolein-based drug-release mechanism potentially has broad applicability with various drugs containing amino or hydroxyl groups (43, 44). Through this mechanism, the DDS can effectively target and deliver conventional anticancer drugs to cancer cells (28). Furthermore, we have developed a doxorubicin prodrug (pro-DOX) using this system to avoid side effects for clinical application (38). In summary, the acrolein-targeting system applied in this study with pro-FTY has previously demonstrated success in various clinical applications.

Our mass spectrometry findings showed that the pro-FTY concentration seemed to be higher in tumors than in blood at d1 after intravenous administration. This finding indicates that pro-FTY can be selectivity activated in cancer cells due to its ability to target acrolein, which is overproduced in cancer cells. In addition, both tissue and blood pro-FTY concentrations had decreased by d3 after administration, whereas breast cancer tissue FTY720 and FTY720-P levels were relatively stable, suggesting that pro-FTY remained longer within the cells, from which FTY720 may have been gradually released. These results suggest that pro-FTY was selectively activated in cancer cells and that released FTY720 may exert an antitumor effect gradually after pro-FTY administration, representing ideal pharmacokinetics.

In contrast, the blood concentrations of FTY720 and FTY720-P were at trace levels because FTY720 was not released (because of the low levels of acrolein in the blood). Because lymphocytopenia occurs at FTY720 concentrations lower than needed for antitumor activity, the lack of observed lymphocytopenia likely indicates that the blood concentration of FTY720 was maintained at very low levels. Although we did not perform detailed pharmacokinetic/pharmacodynamic analysis, stability testing, half-life measurements, or biodistribution experiments for pro-FTY, we previously conducted a series of analysis for our pro-DOX (38). Those experiments revealed that serum protein binding increased significantly (from 68% to >90%) after pro-DOX was derivatized to DOX. Specifically, the hydrophobic protective group in the prodrug enhanced the albumin-binding properties of the drug, thereby improving its serum stability. The stabilized prodrug was selectively activated at the tumor site, in which it reacted with endogenous acrolein to gradually release the active drug. We expect that similar effects could be obtained with FTY720, resulting in high anticancer efficacy.

Given that pro-FTY utilizes a similar DDS, we anticipate that it exhibits pharmacokinetics comparable with previous anticancer prodrugs, such as pro-DOX. However, once activated through reaction with acrolein in cancer cells, the mechanism of action of pro-FTY diverges markedly, based on its specific payload. In contrast to traditional chemotherapeutics (which typically target rapidly dividing cells by disrupting DNA replication, mitosis, or metabolism), FTY720 exerts antitumor effects by modulating S1P signaling. Through this mechanism, FTY720 can promote apoptosis, inhibit angiogenesis, and reshape the tumor-immune microenvironment, thereby offering enhanced selectivity for malignant cells.

Interestingly, FTY720 and pro-FTY consistently suppressed the growth of all breast cancer cell lines tested in this study. FTY720 can inhibit four of five S1P-specific receptors but not S1PR2. Previous data have shown that FTY720 inhibits SphK1, an S1P-producing enzyme. Considering that S1P signaling is essential for cell survival, FTY720 likely inhibits cell growth by blocking S1P receptors and S1P-producing enzymes (45). Cancer cells express elevated levels of S1P-producing enzymes, produce more S1P, and are more dependent on S1P signaling than normal cells, suggesting that anti-S1P signaling therapy may be effective in cancer (15). Previously, we have reported that in inflammatory carcinogenesis and obesity-mediated breast cancer, S1P exacerbates cancer progression through a positive-feedback mechanism mediated by S1PR1 and Stat3 and that FTY720 inhibits tumor growth by blocking this pathway (45, 46). Collectively, these data indicate that after FTY720 is released by the reaction between pro-FTY and acrolein, it inhibits cancer cell survival by blocking the S1P pathway.

Pro-FTY was equally effective against multidrug-resistant breast cancer cell lines and PDOs established from patients with multidrug-resistant breast cancer. As S1P tends to be more highly produced in higher-grade cancers (47), targeting acrolein and S1P with FTY720 may be particularly effective against these cancers. FTY720 inhibits the S1P pathway, representing a completely different mechanism that is used by conventional anticancer drugs, thus rendering pro-FTY an appealing therapeutic for multidrug-resistant cancers. Our results indicate that pro-FTY was not susceptible to cross-resistance and that it might be effective in patients with high-grade breast cancer who exhibit resistance to other anticancer drugs.

Although FTY720 was broadly effective against cancer cells resistant to conventional anticancer drugs, cancer cells can potentially become resistant to FTY720 as well. Resistance to conventional chemotherapy often develops through increased drug efflux or an increased DNA-repair capacity; however, FTY720 resistance is primarily associated with alterations in sphingolipid metabolism and autophagy pathways (48–52). Cancer cells may counteract the effects of FTY720 through mechanisms such as upregulation of SphK1, which facilitates cell survival and proliferation. Decreased sensitivity to FTY720 has been associated with extremely elevated SphK1 expression, which can reactivate prosurvival pathways (48, 49). In addition, FTY720 affects autophagy; in some contexts, FTY720 can induce autophagic cell death under treatment-induced stress, and in other contexts, it may promote cancer cell survival (50–52). Cancer cells can also activate alternative pathways, such as the PI3K–AKT–mTOR axis, to evade FTY720-mediated effects (53). These distinct resistance mechanisms underscore the importance of developing combination strategies and conducting further research to improve the clinical efficacy of FTY720.

To the best of our knowledge, this is the first study to examine the efficacy and side effects of pro-FTY, and certain limitations were involved. Pro-FTY was moderately effective *in vivo*, but more efficient administration methods and combination therapies are needed before clinical application. Doxorubicin and paclitaxel are the most frequently used drugs for treating breast cancer, and the efficacy of pro-FTY720 against corresponding resistant cell lines is of great clinical importance. Notably, because the mechanism of action of pro-FTY differs from those of existing anticancer drugs, additional and synergistic effects can be expected from a combination of drugs. Therefore, developing a multidrug combination therapy including pro-FTY720 is an important issue that should be addressed in the future. Previous data have shown that acrolein is much more highly expressed in cancer tissues than in normal tissues. However, the specific mechanism underlying acrolein production remains unclear. Importantly, this DDS is not limited to FTY720. Rather, it can be applied to other S1P-targeted drugs and may show higher antitumor effects when combined with next-generation targeted therapeutics. We found that acrolein was also highly expressed in gastric cancer, colorectal cancer, and pancreatic cancer; thus, the DDS system may also be applicable to other solid tumors. FTY720 is an oral drug, and effective oral administration of pro-FTY would be very useful clinically. With respect to intravenous administration, oral administration involves more processes before it is absorbed in the gastrointestinal tract, enters the bloodstream, and reaches tumors. Thus, its bioavailability should be studied further to assess the quantity of the drug that can reach tumors, which could highlight the need to explore various chemical modifications.

In conclusion, the novel pro-FTY prodrug (converted to FTY720 through a DDS that targets acrolein, which is highly expressed in cancer) inhibited breast cancer growth but did not cause lymphocytopenia. We believe that this prodrug represents an important step toward the realization of S1P-targeted anticancer therapy, something that has been hindered for many years due to immunosuppressive side effects. We expect that pro-FTY can serve as a novel treatment for difficult-to-treat patients because it is effective against breast cancer that has acquired resistance to multiple conventional anticancer drugs. Because its mechanism of action differs completely from that of conventional chemotherapy, additional and synergistic effects might occur when combined with chemotherapy. Further research is needed to develop the clinical application of pro-FTY.

## Supplementary Material

Supplementary Figure 1 Hematoxylin and eosin (HE)-stained specimens of heart, kidney, liver, lung, and spleen in control mice, and FTY720 or pro-FTY-treated mice.
