# Supplementary figures and images for: An Acrolein-Based Drug Delivery System Enables Tumor-Specific Sphingosine-1-Phosphate Targeting in Breast Cancer without Lymphocytopenia
Source: Cancer Res Commun. 2025 Jun 18;5(6):981–93. doi: 10.1158/2767-9764.CRC-25-0023 (PMC12174973; doi:10.1158/2767-9764.CRC-25-0023)

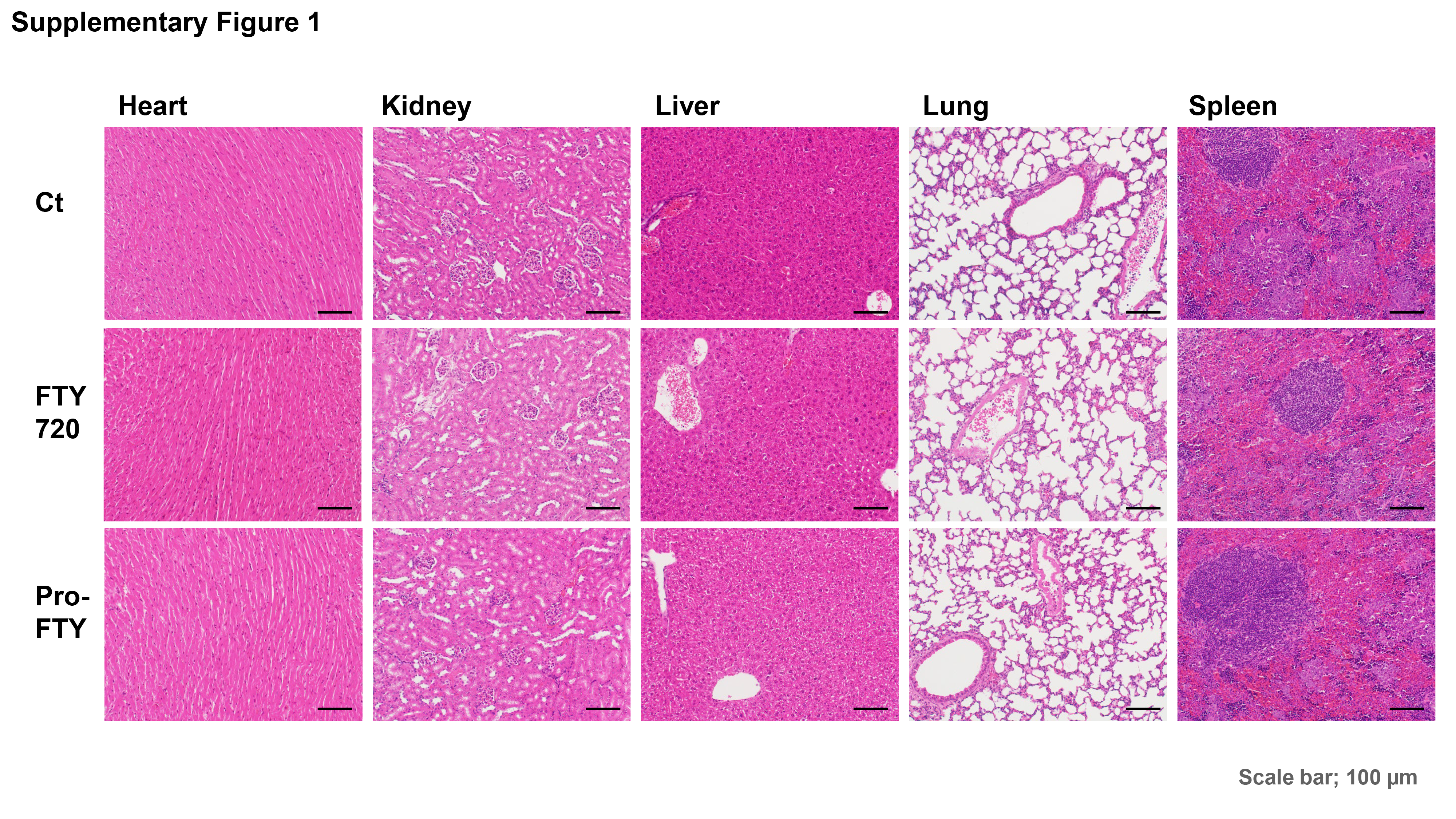

Supplement: Supplementary Figure 1 — Hematoxylin and eosin (HE)-stained specimens of heart, kidney, liver, lung, and spleen in control mice, and FTY720 or pro-FTY-treated mice. [file crc-25-0023_supplementary_figure_1_suppsf1.png]
